# Surface and Mineral Changes of Primary Enamel after Laser Diode Irradiation and Application of Remineralization Agents: A Comparative In Vitro Study

**DOI:** 10.3390/children11091069

**Published:** 2024-08-30

**Authors:** Mihaela-Valentina Cîrdei, Mădălin-Marius Margan, Roxana Margan, Alexandra Ban-Cucerzan, Ion Petre, Iosif Hulka, Razvan Mihai Horhat, Darinca Carmen Todea

**Affiliations:** 1 Department of Oral Rehabilitation and Dental Emergencies, Faculty of Dentistry, Victor Babes University of Medicine and Pharmacy, 300041 Timișoara, Romaniatodea.darinca@umft.ro (D.C.T.); 2 Department of Functional Sciences, Discipline of Public Health, Victor Babes University of Medicine and Pharmacy, 300041 Timișoara, Romania; 3 Center for Translational Research and Systems Medicine, Faculty of Medicine, Victor Babes University of Medicine and Pharmacy, 300041 Timișoara, Romania; 4 Department of Microbiology, Discipline of Hygiene, Victor Babes University of Medicine and Pharmacy, 300041 Timișoara, Romania; 5 Center for Studies in Preventive Medicine, Faculty of Medicine, Victor Babes University of Medicine and Pharmacy, 300041 Timișoara, Romania; 6 Department of Animal Production and Veterinary Public Health, Faculty of Veterinary Medicine, University of Life Sciences “King Mihai I” from Timişoara, 300645 Timișoara, Romania; alexandracucerzan@usvt.ro; 7 Department of Functional Sciences, Discipline of Medical Informatics and Biostatistics, Victor Babes University of Medicine and Pharmacy, 300041 Timișoara, Romania; 8 Research Institute for Renewable Energies, Politehnica University Timișoara, No. 138, Gavril Musicescu Street, 300774 Timișoara, Romania; iosif.hulka@upt.ro; 9 Department of Restorative Dentistry, Faculty of Dentistry, Victor Babes University of Medicine and Pharmacy, Digital and Advanced Technique for Endodontic, Restorative and Prosthetic treatment Research Center (TADERP), 300041 Timișoara, Romania; horhat.razvan@umft.ro; 10 Interdisciplinary Research Center for Dental Medical Research, Lasers and Innovative Technologies, 300041 Timișoara, Romania

**Keywords:** low-light laser therapy, DIAGNOdent, fluoride varnish, energy-dispersive X-ray spectroscopy (EDX), temporary enamel, SEM

## Abstract

Objectives: The purpose of this study is to evaluate the remineralization potential of primary teeth enamel after being exposed to different laser diode therapies. Methods: Ninety-six vestibular primary teeth enamel samples were divided into eight groups (*n* = 12) with varying treatments: control (G1), CPP-ACP-fluoride varnish (G2), diode lasers at 980 nm (G3), 808 nm (G4), 450 nm (G5), 980 nm + CPP-ACP-fluoride varnish (G6), 808 nm + CPP-ACP-fluoride varnish (G7), and 450 nm + CPP-ACP-fluoride varnish (G8). Each sample was assessed using a DIAGNOdent^®^ (KaVo Dental, Biberach, Germany), at baseline, post-treatment, and post-pH cycle remineralization. SEM imaging was performed before and after treatment and following the pH cycle. Results: The results indicated that the 980 nm and 808 nm diode lasers, both alone and in combination with CPP-ACP-fluoride varnish, either maintained or increased the calcium (Ca) weight percentage (Wt%) in the enamel. The 980 nm diode laser combined with CPP-ACP-fluoride varnish (G6) showed a significant increase in Ca Wt%, suggesting a strong remineralization effect. Similarly, the 808 nm diode laser alone (G4) also promoted a substantial increase in Ca Wt%. In contrast, the 450 nm diode laser, whether applied alone or in combination with CPP-ACP-fluoride varnish, resulted in a lower Ca Wt% and an increase in phosphorus (P) Wt%. Most groups, except for the CPP-ACP-fluoride varnish alone (G2), demonstrated an increase in P Wt%, indicating a complex interaction between laser therapy and enamel remineralization. Conclusions: The combined use of laser therapy with CPP-ACP-fluoride varnish significantly enhanced the remineralization of temporary teeth enamel. The 980 nm diode laser + CPP-ACP-fluoride varnish showed the most pronounced improvement in remineralization, while the 808 nm diode laser alone also effectively increased calcium solubility. These findings suggest that higher-wavelength diode lasers, particularly when combined with remineralizing agents, can effectively enhance the mineral content of primary teeth and promote enamel remineralization.

## 1. Introduction

Demineralization and initial carious lesions remain prevalent among children despite improvements in dental care [1,2,3,4]. Studies indicate a global rise in dental caries in both temporary and permanent teeth, linked to public health concerns and socioeconomic inequalities [5]. A caries develops from an imbalance between demineralization and remineralization processes, influenced by various factors [1,6,7]. White spot lesions are early indicators of caries; untreated, they can become cavitated due to lower hardness compared to intact enamel [1,8]. However, demineralized enamel can regenerate under favorable conditions, reducing dentinal hypersensitivity and caries progression [9,10,11]. Enhancing the concentration of calcium or fluoride in oral fluids can promote remineralization and repair lesions, particularly in the early stages of enamel caries, where fluoride-containing products are especially effective [3,9,11,12,13].

Dental caries result from interactions among salivary and genetic factors, carbohydrates, microbial biofilm, and tooth structure. The development of caries involves alternating phases of demineralization and remineralization, with pathological factors favoring caries and protective factors promoting remineralization [13,14]. Bacterial acids cause the dissolution of mineral ions, forming enamel hydroxyapatite crystals, while saliva plays a key role in restoring pH levels after carbohydrate intake [11,12].

Calcium phosphate-based remineralization techniques, particularly those using casein phosphopeptide–amorous calcium phosphate (CPP-ACP), have shown promise in early lesion repair [1,10]. Recent research has focused on non-invasive remineralization techniques, including the use of fluoride, calcium, and phosphate ions in various forms [15,16,17,18,19,20,21]. Among these, CPP-ACP demonstrates significant anticariogenic properties, supported by biomimetic regeneration technologies and systems that enhance fluoride effectiveness [22,23,24,25,26,27,28].

Laser technology has emerged as an adjunct preventive measure against carious lesions, with various wavelengths (e.g., 810–830 nm, 940 nm, 980 nm, and 1064 nm) showing effectiveness in altering enamel microstructure and reducing its solubility [29,30,31,32]. Lasers, such as Er:YAG, Nd:YAG, and CO_2_, have been studied for their ability to enhance enamel microhardness and decrease enamel permeability, often producing a synergistic effect when combined with topical fluoride treatments. This combination has been shown to significantly enhance fluoride uptake and reduce enamel breakdown rates [33,34,35,36,37,38]. Additionally, laser treatments can decrease *Streptococcus mutans* bacteria in the mouth, contributing to caries prevention [39,40,41,42,43]. The application of laser light not only strengthens enamel but also offers patient comfort by reducing treatment times and eliminating vibrations [30,37,38,39].

Thus, maintaining a balance between demineralization and remineralization is crucial for preventing and reversing early carious lesions. Advances in remineralization techniques, including the use of CPP-ACP and laser technology, offer promising strategies for enhancing oral health and reducing the incidence of dental caries [1,11,21,22,23,24,29,30,31,32,33,34,35,36,40,41,42,43].

The purpose of this study is to assess and contrast the effects of three low-light laser wavelengths on the temporary enamel surface and evaluate the composition of mineral components using SEM and EDX spectroscopy analysis. This research is relevant for its potential to reduce the impact of bacterial lesions on enamel by altering its chemical structure and evaluating the effectiveness of laser technology as a preventive treatment in pediatric and restorative dentistry.

We evaluated the following null hypotheses for this study:

1. There will be no alterations to the enamel surface following treatment with or without laser and after the pH cycle;
2. The percentage of chemical elements in the measured regions will remain unchanged following treatment with or without laser and after the pH cycling;
3. The three wavelengths will not have a distinct impact on the chemical structure of the enamel.

## 2. Materials and Methods

This in vitro study was approved by the local research ethics committee of the University of Medicine and Farmacy Victor Babes Timisoara (Project Nr. 84/25.04.2022 rev). The experimental parts were conducted in the Faculty of Dentistry, in collaboration with the Department of Animal Production and Veterinary Public Health, University of Life Sciences “King Mihai I” from Timişoara, Romania, the University of Veterinary Medicine Timisoara, and with the Research and Renewable Energies, Timisoara. The research procedure was thoroughly explained to all participants, who then provided informed consent by signing a permission form authorizing the use of their data and samples for scientific research purposes.

Phase one of the investigation involved clinical tooth selection; phase two involved in vitro tooth preparation, experimental techniques, and morphological characteristics and chemical component analysis of the enamel.

### 2.1. Tooth Selection and Preparation Phase

A total of 120 temporary teeth were initially collected for this investigation; however, only 96 vestibular enamel teeth samples (16 molars, 10 incisors, 24 lateral incisors, 30 central incisors, and 16 canines) were selected after applying inclusion and exclusion criteria. The inclusion criteria required primary teeth (incisors, canines, molars) without vestibular enamel defects, no prior procedures, stage 2 or 3 physiological root resorption, and no visible pits or fissures. Teeth with visible pits and fissures, previous dental work, interproximal cavity lesions, or endodontic treatment were excluded.

Following extraction, the teeth were cleaned of any remaining soft tissues and preserved in a physiological serum, with the solution replaced every 24 h until further processing. Each sample was then cleaned using an ultrasonic scaler (EMS miniPiezon, SA CH-1260, Nyon, Switzerland), brushed with non-fluoridated toothpaste (Cleanic^TM^ Prophylaxis Paste, KerrHawe SA, 6934 Bioggio, Switzerland), and rinsed with distilled water.

**Figure 1:**
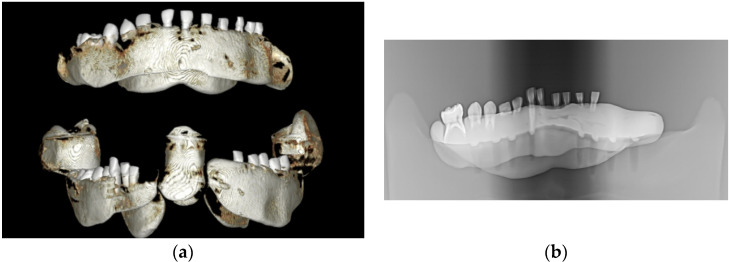
The images captured using cone beam computed tomography (CBCT) and panoramic X-ray depict selected teeth from the study groups: (**a**) CBCT imaging and (**b**) panoramic X-ray imaging.

All samples were mounted on a plastic mandible for external radiographic examination using 3D or 2D X-rays (Figure 1), ensuring no signs of demineralization or other dentine lesions were present. The clinical steps of the study were documented and photographed using an iPhone XS MAX (Apple, Cupertino, CA, USA).

**Figure 2:**
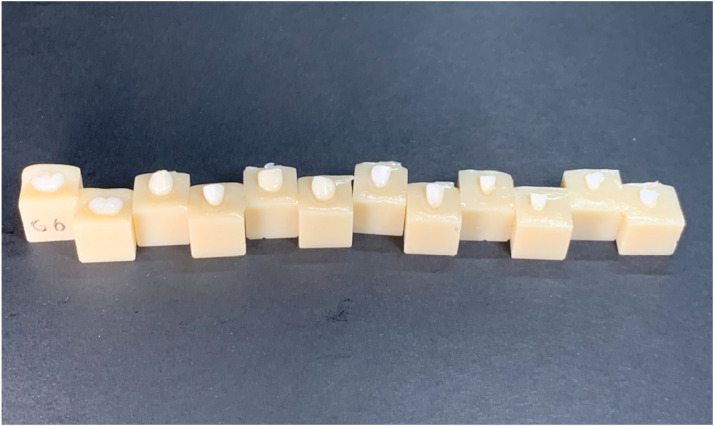
The bis-acrylate composite blocks of one of the temporary enamel study groups.

The enamel surface samples of the temporary teeth were embedded into bis-acrylate composite blocks (LUXATEMP Star DMG A2 REF 110912, DMG, Hamburg, Germany), ensuring that the enamel surface remained exposed (2 × 2 mm). Two layers of clear nail polish were applied to completely cover the dentine surfaces and any additional areas not involved in the study. The teeth were then coded and randomly assigned to eight experimental groups, with each group containing an equal number of teeth and a balanced representation of molars, canines, upper central incisors, lateral incisors, and lower central incisors (Figure 2). This study comprised the following eight groups: (1) control group (G1); (2) fluoride varnish (MI-fluoride varnish—CPP-ACP) (G2); (3) diode laser 980 nm (G3); (4) diode laser 808 nm (G4); (5) diode laser 450 nm (G5); (6) diode laser 980 nm + MI-fluoride varnish (CPP-ACP) (G6); (7) diode laser 808 nm + MI-fluoride varnish (CPP-ACP) (G7); and (8) diode laser 450 nm + MI-fluoride varnish (CPP-ACP) (G8).

### 2.2. Experimental Techniques

#### 2.2.1. DIAGNOdent^®^ Pen

**Figure 3:**
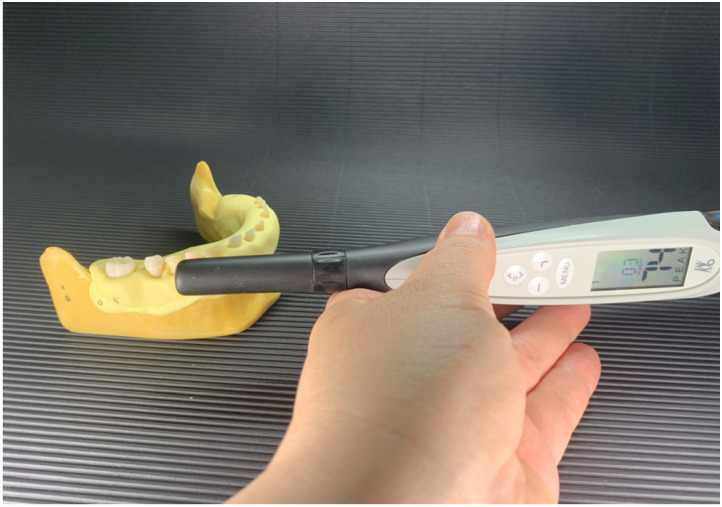
Recording of the initial values of the temporary enamel surface of one of the sample groups with the DIAGNOdent^®^.

In accordance with the manufacturer’s instructions, each tooth sample was assessed using a cylindrical sapphire-fiber tip specifically designed for smooth surfaces, employing the Laser Fluorescence (LF) DIAGNOdent^®^ Pen 2190 (KaVo, Biberach, Germany). Calibration was performed using the ceramic standard before each measurement. Each block was dried with a paper tissue and then air-dried for 5 s. Recalibration was conducted after every 10 scanned sample surfaces. Fluorescence measurements were recorded at three stages: before any treatment, following treatment with or without laser application, and after each day of the 7-day pH cycle (Figure 3).

#### 2.2.2. Applied Treatments

1. Topical fluoride varnish containing calcium and phosphate, specifically fresh mint 0.44 g (0.4 mL) (MI Varnish™ by GC Corporation, Tokyo, Japan), was applied to the enamel surface of Group 2 (G2) using a microbrush. For Groups 6 (G6), 7 (G7), and 8 (G8), the MI fluoride varnish was administered following laser irradiation. A comprehensive overview of the treatments administered across all eight groups is provided in Table 1. Following each experimental procedure, the samples were immersed in artificial saliva for 24 h until the subsequent step. The composition of the artificial saliva, as described by Serdar et al. [44], included 0.4 g of NaCl, 1.21 g of KCl, 0.78 g of NaH_2_PO_4_·2H_2_O, 0.005 g of Na_2_S·9H_2_O, 1 g of CO(NH_2_)_2_, and 1000 mL of distilled deionized water. After the 24 h immersion period, the hardened varnish was removed from the tooth surface through brushing and scaling to simulate regular oral hygiene practices.

2. For the laser groups, the parameters used were different according to the laser wavelength. All laser procedures were performed by the same operator, who scanned uniformly over the enamel surface to ensure complete coverage of the chosen region. The treatments for the laser groups were as follows: Groups 3 (G3) and 6 (G6) were treated with a 980 nm diode laser (KaVo GENTLEray 980 Diode Laser, Kaltenbach & Voigt GmbH, Biberach, Germany). The procedure involved the use of a 300 μm optic fiber at a wavelength of 980 nm, with an output power of 1 W, applied for 60 s in continuous contact mode without water. For Group 6 (G6), following laser irradiation of the enamel surface, MI-CPP-ACP fluoride varnish was applied according to the manufacturer’s instructions.
3. For Groups 4 (G4) and 7 (G7), the treatment involved 808 nm diode laser irradiation under the following conditions: A 400 μm optic fiber was employed at a wavelength of 808 nm, with a peak power of 2.5 W and an average power of 0.42 W (WISER 3 DOCTOR SMILE, P. IVA, by Lambda S.p.A., Via dell’Impresa, Brendola (VI), Italy). The procedure featured a frequency rate of 8.33 kHz, with parameters of 0.1 W/cm^2^ delivering 2 J of energy, amounting to a total energy of 8.3 J per tooth sample and 75.1 J per procedure. The laser was operated in continuous-wave, pulsatile, and contact modes, with an exposure time of 20 s per sample and without the use of water for scanning the tooth surface. After laser irradiation of the enamel surface in Group 7 (G7), MI-fluoride varnish was subsequently applied.
4. For Groups 5 (G5) and 8 (G8), the treatment involved 450 nm laser irradiation using a 400 μm optic fiber with a wavelength of 450 nm (WISER 3 DOCTOR SMILE, P. IVA, by Lambda S.p.A., Via dell’Impresa, Brendola (VI), Italy). The procedure was conducted with a peak power of 0.4 W, an average power of 0.07 W, and a frequency rate of 8.33 kHz. The parameters included 0.1 W/cm^2^, delivering 2 J of energy, resulting in a total energy of 3.45 J per tooth sample. The laser was operated in continuous, pulsatile, and contact modes with an exposure time of 30 s per sample, without the use of water for scanning the tooth surface. Following laser irradiation in Group 8 (G8), MI-CPP-ACP fluoride varnish was applied.

After completing the treatments specific to each group, all samples underwent a 7-day *in vitro* pH cycling process as previously described by Cate and Duijsters [45]. This process began for all samples, where they were immersed for 6 h in a demineralization solution. The solution composition cited by Serdar et al. [44], was adjusted to achieve a pH of 4.5 by modifying the amount of 1 M KOH. The reactive components included 2 mM CaCl_2_, 2.2 mM NaH_2_PO_4_, 0.05 M CH_3_COOH, and 0.05 M KOH, with a maintained temperature of 37 °C. To ensure the accuracy and consistency of the experimental conditions, the solutions were replaced daily, and new solutions were prepared in the laboratory every three days [44].

### 2.3. Evaluation Methods

#### 2.3.1. The DIAGNOdent^®^

An LF pen was employed to assess potential changes in the temporary enamel surface values throughout the study. Initially, the enamel surface was evaluated using the DIAGNOdent^®^ Pen prior to any treatment procedures. After the treatments specific to each group were applied, a second DIAGNOdent^®^ Pen measurement was recorded. Before entering the 7-day pH cycling process, which simulates remineralization, another set of measurements was taken with the DIAGNOdent^®^ Pen.

Following the exposure to the demineralization solution for the prescribed duration, the samples were thoroughly rinsed with distilled water, dried, and then reassessed using the DIAGNOdent^®^ pen to obtain a new set of values for the temporary enamel surface. Subsequently, the samples were immersed for 18 h in a remineralization solution composed of 1.5 mM CaCl_2_, 0.9 mM NaH_2_PO_4_, and 0.15 M KCl at a pH of 7.0. After the 18 h immersion period in the remineralization solution, the samples were again rinsed with distilled water, dried, and subjected to a new set of measurements using the DIAGNOdent^®^ pen. This process was repeated daily over a 7-day period, with the samples undergoing both demineralization and remineralization cycles. Notably, after the fifth day of the pH cycle, the samples were only immersed in the remineralization solution during the sixth and seventh days.

Throughout the 7-day pH cycle, two DIAGNOdent^®^ scans were performed each day, and the resulting values were recorded, except for the last two days when only one scan was conducted. The repeated use of DIAGNOdent^®^ scanning served as an ongoing evaluation method to monitor changes in the enamel surface. Figure 3 illustrates a scanning evaluation performed on one of the temporary enamel surface structures prior to the commencement of any study procedures.

#### 2.3.2. Scanning Electron Microscopy (SEM) and Energy-Dispersive X-ray Spectroscopy (EDX) Analysis

The structure and elemental composition of the samples under examination were ascertained by applying the scanning electron microscopy—SEM (Quanta FEG 250, FEI, Hillsboro, OR, USA) imaging in conjunction with energy-dispersive spectroscopy (EDS/EDAX). The chemical analysis was performed by energy-dispersive X-ray spectroscopy (EDX with Apolo SSD detector; EDAX Inc., Mahwah, NJ, USA) to reveal the elemental composition for all the groups before and after the treatment with or without laser or in combination, and after pH cycle remineralization at the Research Institute for Renewable Energies, Politehnica University Timisoara.

One sample from each group was subjected to the same treatment as the others, with the additional step of being dried for 24 h under vacuum conditions prior to EDAX/EDS (energy-dispersive X-ray Spectroscopy) measurements. The SEM (scanning electron microscope) was operated in low vacuum mode to prevent sample charging, using a cathode voltage of 15 kV and a backscattered electron detector (BSD). The integrated EDX (energy-dispersive X-ray) system functioned as a core component of the SEM Quanta FEG 250 instrument.

For the analysis, the teeth were mounted on an aluminum stage using carbon tape, and after sealing the SEM chamber, the EDAX examination commenced with an accelerated voltage range of 1–80 kV. This process was conducted to determine the elemental structure and composition of the enamel in each group. Elemental levels were quantified and expressed in both weight percentage (Wt%) and atomic percentage (At%) for key elements including calcium (Ca), fluoride (F), phosphorus (P), aluminum (Al), and oxygen (O). The primary focus was on calcium and phosphorus, as their ratio is particularly important given that numerous studies have demonstrated the influence of these elements on enamel microhardness [40,46,47,48].

The SEM and EDAX/EDS analyses were repeated following the 7-day pH cycle to evaluate the differences in remineralization across the groups.

### 2.4. Statistical Analysis

Data collection, management, and analysis were conducted using MediFlux™ software v1.1 (developed by Originis™, Amsterdam, The Netherlands). Statistical computations were conducted using R version 4.2.0 (R Foundation for Statistical Computing, Vienna, Austria). Data manipulation was handled with the R base package, while statistical tests between numerical variables were performed using the R stats package. Graphs and plots were generated using the “ggplot2” package (version 3.5.1).

Continuous variables were expressed as mean ± standard deviation. Paired *t*-tests were conducted to compare changes within groups before and after treatments, helping to assess the impact of different conditions on the variables of interest.

ANOVA (analysis of variance) was used to determine whether there are statistically significant differences between the means of three or more independent groups. When significant differences were found, post-hoc tests, such as the Tukey HSD test, were used to make detailed comparisons between specific pairs of groups. 

## 3. Results

### 3.1. Scanning Electron Microscopy (SEM)

**Figure 4:**
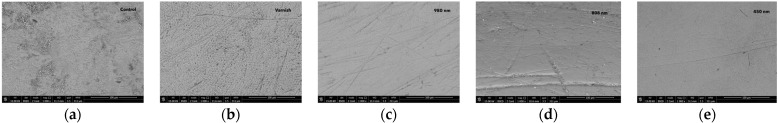
The images recorded by scanning electron microscopy for different groups: (**a**) control group; (**b**) after MI-fluoride varnish treatment; (**c**) after 980 nm laser irradiation; (**d**) after 808 nm laser irradiation; and (**e**) after 450 nm laser irradiation.

**Figure 5:**
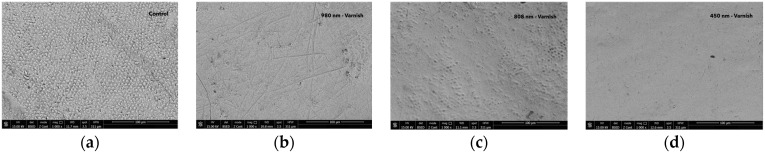
The images recorded by scanning electron microscopy for different groups: (**a**) control group after pH cycle; (**b**) 980 nm laser + MI-fluoride varnish group after pH cycle remineralization; (**c**) 808 nm laser + MI-fluoride varnish group after pH cycle remineralization; and (**d**) 450 nm laser + MI-fluoride varnish group after pH cycle remineralization.

Scanning electron microscopy at ×1000 magnification (Figure 4 and Figure 5) revealed the surface topography and morphology of the control enamel region, the MI-fluoride varnish group, and the treatment groups using 980 nm, 808 nm, and 450 nm laser diodes. The scanning electron microscopy (SEM) and energy-dispersive X-ray spectroscopy (EDX) mean data are presented below (Figure 4).

The enamel surface of the control group showed signs of unevenness, including small holes, roughness, and dirty surfaces. For group (G2), after the varnish treatment, the enamel surface showed signs of unevenness, including small holes, roughness, and indentations. 

After 980 nm diode laser irradiation, the appearance of the enamel was characterized by a smooth, relatively uniform, and crack-free surface with small irregularities and some indentations. 

After 808 nm diode laser irradiation, the enamel surface showed signs of unevenness, including some larger indentations. 

After laser irradiation with 450 nm diode laser, the enamel surface appeared smooth, largely homogeneous, devoid of cracks, and had minor imperfections and unevenness.

For the control group, the surface of the enamel showed large prism roughness after the pH cycle. After 980 nm diode laser irradiation and MI-fluoride varnish, the surface of the temporary enamel was smoother, largely homogeneous, devoid of cracks, and had minor imperfections following pH cycling. After 808 nm diode laser irradiation and MI-fluoride varnish, the surface of the enamel was relatively smooth, largely homogeneous, presenting tiny holes, devoid of cracks, and had minor imperfections following pH cycling.

Finally, in the laser 450 nm and MI-fluoride group, the surface of the temporary enamel varnish was smooth, uniform, largely homogeneous, and crack-free surface following pH cycling. 

The findings revealed that the three laser-irradiated groups, treated exclusively with laser wavelengths of 980 nm, 808 nm, and 450 nm, exhibited a smoother temporary enamel surface compared to both the fluoride-treated group and the untreated control, as illustrated in Figure 4. Following the pH cycle remineralization period, the groups that received a combination of laser diode treatment and subsequent fluoride varnish application demonstrated an even smoother enamel surface structure. These treated samples showed a significant reduction in surface roughness. The SEM images provided a clear and detailed visualization of the effects of the different laser treatment approaches, as shown in Figure 4 and Figure 5.

### 3.2. DIAGNOdent^®^ Analysis

**Figure 6:**
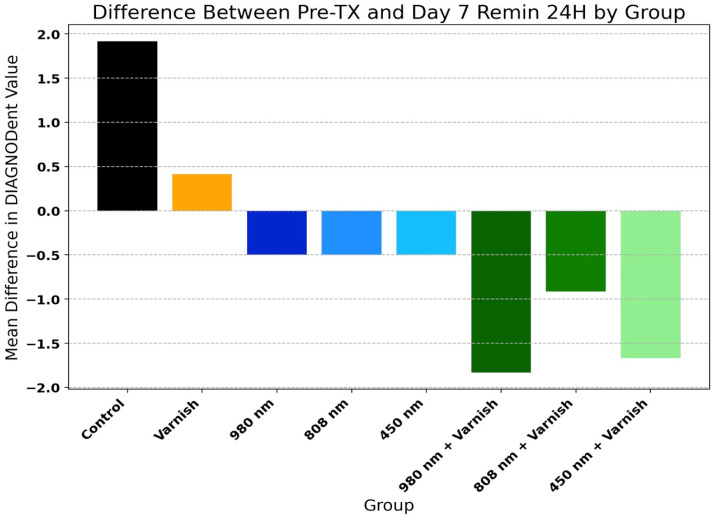
Difference between Pre-TX (pre-treatment) and day 7 pH 24 h (day 7 remineralization at 24 h) by group.

Table 2 and Figure 6 present the mean values of DIAGNOdent^®^ scores of temporary enamel at initial scanning and in the last day of pH cycle remineralization for the study groups. There was a highly significant decrease in the mean values of DIAGNOdent^®^ enamel scores in all groups, with the exception of the varnish and laser diode 980 nm groups. This proves that all three lasers used and in combination with the remineralizing agent used in this study are effective in remineralizing the enamel surface. 

Figure 6 displays the comparison of relative improvement of DIAGNOdent^®^ scores among study groups (one-way ANOVA: F = 25.03, *p* < 0.001).

### 3.3. EDX Chemical Analysis

The results of this study demonstrated statistically significant differences in the effects of various laser treatments—specifically the 980 nm diode laser, the 808 nm diode laser, and the 450 nm laser diode—on the concentrations of calcium (Ca) and phosphorus (P) following the pH cycle of remineralization. Based on the findings, the 808 nm diode laser exhibits a greater efficacy in augmenting the weight percentage (Wt%) of calcium and phosphorus than the 980 nm diode laser, particularly in relation to the calcium weight percentage after the pH cycle remineralization treatment.

Notably, after treatment with the 450 nm laser diode during the pH cycle, while there was a reduction in the weight percentage of calcium, there was a slight increase in the weight percentage of phosphorus. Furthermore, exposure to both the 808 nm diode laser in conjunction with MI-fluoride varnish and the 450 nm laser diode with MI-fluoride varnish resulted in decreased calcium levels; however, these treatments led to a comparatively greater increase in the weight percentage of phosphorous than that observed with the 450 nm laser diode alone.

In contrast, treatment with the combination of the 980 nm diode laser and MI-fluoride varnish, along with treatment with the 808 nm laser diode, yielded increases in both calcium and phosphorus weight percentages. Our hypothesis was substantiated through the application of an independent *t*-test. 

Table 3 highlights the Tukey HSD results for mean values of EDX weight percentage values between each laser group with its correspondent laser with the MI-CPP-ACP fluoride varnish group, with the Varnish and the Control groups.

Table 4 presents the comparative weight percentages of mineral elements across the various study groups, both after the treatment and after the pH cycle, with statistical significance established via ANOVA (*p* < 0.001). The data indicate a notable increase in calcium (Ca) levels following both irradiation treatments, specifically the 808 nm diode laser and the 980 nm laser diode in conjunction with fluoride varnish. Notably, the application of the 980 nm laser diode alone maintained a consistent percentage of calcium after pH cycling remineralization.

In terms of phosphorus (P), increases were observed across all laser irradiation groups, regardless of the application of MI-fluoride varnish. Additionally, the weight percentage (Wt%) of oxygen (O) was significantly higher in all treatment groups compared to baseline values prior to treatment. Conversely, the weight percentage of carbon (C) exhibited a general decline across all treatment groups, with the exception of the group subjected to the 450 nm laser diode. No statistically significant differences were detected in the mean values of other mineral elements, including sodium (Na), magnesium (Mg), aluminum (Al), silicon (Si), and chlorine (Cl), following the various treatments administered.

Table 5 shows the Tukey HSD results for Ca/P ratios between groups, summarizing just the significant findings where just true results reject the null hypothesis.

**Figure 7:**
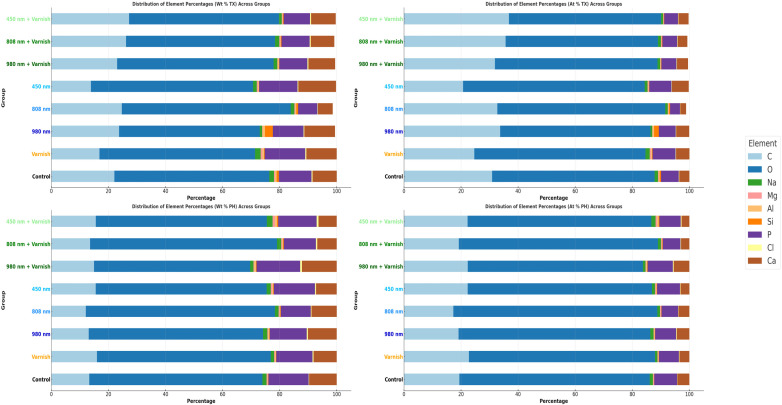
Comparative mean weight percentage (Wt%) and mean atomic weight (At%) of mineral elements between study groups; TX—after treatment; PH—after pH cycle remineralization.

Figure 7 shows the mean comparative weight percentage (Wt%) and mean atomic weight percentage (At%) of mineral elements after treatment and after the pH cycle between study groups.

**Figure 8:**
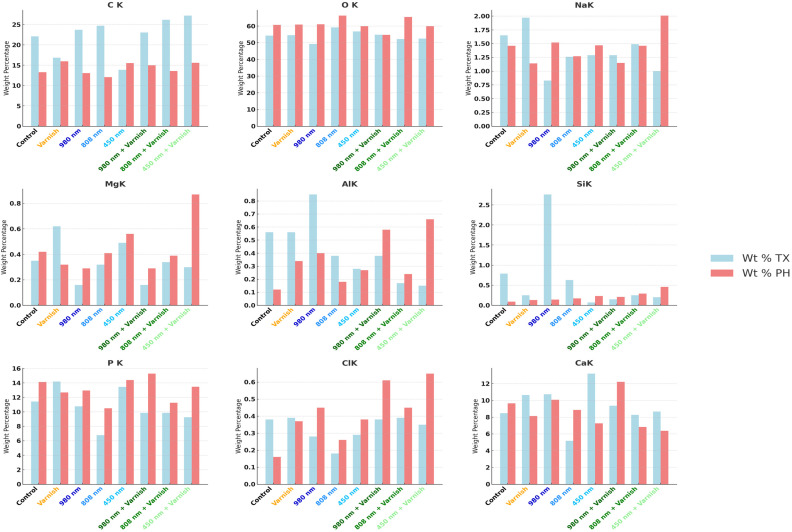
Comparative mean weight percentage (Wt%) of individual mineral elements in study groups; TX—after treatment; PH—after pH cycle remineralization.

Similarly, Figure 8 illustrates the mean weight percentage (Wt%) after treatment and after the pH cycle focused on individual mineral elements. 

## 4. Discussion

Prevention should always be a fundamental priority in oral–dental health, especially for our young patients that are confronted from an early age with demineralization, white spots, or incipient lesion caries [44].

In the current study, the new diode laser wavelengths of 808 nm and 450 nm diode laser were chosen, with consideration given to the significant results shown in studies related to the 980 nm laser diode. It was aimed at determining whether similar results would be obtained for the new 808 nm laser diode and the 450 nm laser diode. Certain limitations exist within the research model, including the inability to predict the long-term effects of varnish or laser therapy and the unclear impact of subsequent acid attacks. Consequently, additional abrasive forces were not included in the experimental model to simulate greater resistance of the enamel.

Given that demineralization remains a reversible process at this stage of lesion development, where cavitation has yet to occur, it is imperative to investigate the efficacy of novel preventative interventions [49]. Among these, fluoride treatment stands out as the most effective method for augmenting the enamel’s resistance to demineralization. Furthermore, high-intensity laser applications have demonstrated a capability to influence the solubility of enamel, thus reducing the rate of mineral loss by altering its crystalline structure. Additionally, low-level lasers have exhibited potential in enhancing the resilience of enamel under acidic conditions [50,51,52,53,54,55].

The diode laser family has expanded significantly since they were first introduced to the medical field by being applied to different pathologies since they are smaller and less expensive. Diode lasers are the best option in pedodontics, restorative, and orthodontic procedures; thus, the continuous-wave mode and non-contact mode are favorable for their application [31].

Despite these constraints, the model proves valuable by isolating the effects of three types of laser treatment against a 7-day pH cycle remineralization, which is particularly relevant for preventive strategies. This aids in refining laser treatment protocols by offering insights into the appropriate frequency of laser application, parameters, and the underlying mechanisms of action. Consequently, it enables a thorough investigation into both the prevention of enamel softening and the potential for rehardening [44,56].

Because academic literature has reported that CPP-ACP-fluoride varnish (MI-fluoride varnish) has the best results in increasing structural dentine resistance when combined with different types of lasers, specifically laser diode 980 nm and Er:YAG, Nd:YAG, the current study focused on the three types of lasers and only one reinforcement agent. The purpose was to examine whether there would be any changes in the morphological structure of the enamel and what would happen with the Ca/P ratio after the EDX spectrometry with the assistance of two different low lasers at 808 and 450 nm, which are fairly new in the use of hard tissues [44,56,57].

Fluoride can convert to calcium fluoride and become a part of the enamel structure if it is present during the acid challenge [58,59]. According to reports, calcium fluoride functions as a reservoir for fluoride and can significantly lessen enamel demineralization during the acid challenge [59]. The use of fluoride is one of the most studied, known, and effective methods to prevent dental caries [1,13,25,27,35]. Much of the success attributed to fluoride is due to its capacity of reversing the beginning and progression of caries [17]. Even though the water can control the increase of the temperature, in our study, we did not use water cooling during low laser irradiation because it is well known that in the event of an acid challenge, the demineralization of the enamel is greater if the surface of the enamel is in direct contact with water [60]. 

Because of the increased surface roughness, it is thus questioned if these factors, which have been demonstrated to alter the chemical structure of the enamel and to strengthen its acid resistance, might also aid in the adherence of bacteria to the enamel [61]. Also, the bacteria can lead to enamel demineralization through the acids that they produce [59]. Saliva’s buffering ability and propensity for remineralization can help maintain the mineral balance; hence, it is anticipated that both in situ and in vivo situations may result in a less significant mineral loss [14,21,59]. 

There is a considerable number of studies in the area of prevention [1,5,13,18,25,27,35] and treatment of white spot lesions [10,12,29]. One way to assess changes in enamel is by surface microhardness analysis [40]. 

The degree of lightness was found to diminish more after the severe remineralization regimen (T3), which included fluoride therapy with or without laser application [13]. The current study is the first attempt to demonstrate that the two 808 nm and 450 nm diode laser irradiations, before the MI-fluoride varnish application, are effective in improving the microhardness of the enamel surface. 

It is believed that the increased microhardness following laser irradiation is related to the ultrastructural changes, including crystal size growth and recrystallization of porous enamel, as a result of high temperature rise at the surface [62].

Diode laser alone was not beneficial for lesion prevention, according to Santaella et al. [63], who also discovered that materials comprising fluoride, calcium, and phosphate were more effective than diode laser alone. Diode laser irradiation enhanced the absorption of ions by the enamel structure, particularly fluoride, as demonstrated by González-Rodríguez et al. [64] and Vitale et al. [65]. One possible explanation is that the laser’s thermal effects make the tooth surface harder, which in turn allows more fluoride to penetrate and stay on the surface. Their outcomes validated our conclusions.

They confirmed our results on the greater effectiveness of phosphate, calcium, and fluoride ions, but their conclusions about the ineffectiveness of the diode laser by itself did not match our findings. According to Kato et al. [66], a diode laser by itself was unable to reduce the solubility of enamel.

There was an improvement in enamel resistance to acid attack in the laser-fluoride and laser groups as compared to the fluoride group, according to Apel et al. [67]. Evidence by Rodrigues et al. [68] suggests that the demineralization resistance of enamel can be enhanced by combining CO_2_ laser with fluoride ions. According to their findings, the laser outperformed fluoride in preventing tooth decay. One possible explanation is that they used fluoridated toothpaste, which contains a lower quantity of fluoride and is hence less effective than varnishes.

The relationship between light and certain enamel components and the efficacy of laser irradiation as a preventative therapy for caries is significant [45,69]. The tissue’s ability to absorb laser light is a crucial characteristic. Similar to bone, dental hard substances are made up of three main components: organic matrix such as proteins or collagen, inorganic compounds like hydroxyapatite and TCP (enamel: 96%; dentin: 69%; bone: 46%), and water (enamel: 2.5%; dentin: 13.5%; bone: 32%). The predominant inorganic component is hydroxyapatite Ca10(PO_4_)6 (OH)_12_. The primary absorption peak of hydroxyapatite occurs at a wavelength of 9.6 pm [70,71]. Fluoride aids in remineralization by increasing fluoride levels in the enamel, forming fluorapatite, which is more resistant to acid attacks than hydroxyapatite. It also inhibits bacterial metabolism, thereby reducing acid production. Consequently, this factor influences how we approach preventive measures for primary enamel. Research has demonstrated that the structural differences between primary and permanent enamel impact their demineralization rates, highlighting the importance of these differences in designing effective dental treatments [72,73].

Disruptions in the balance of mineralization due to factors like cariogenic bacteria, external agents, heat, cold, and erosive foods and drinks can lead to the loss of mineralized tooth tissues and dental decay. This issue is especially significant for primary teeth, which have thinner and less mineralized enamel compared to permanent teeth (with mineralization levels of 80.6% in deciduous enamel versus 89.7% in permanent enamel) and an outer layer known as aprismatic enamel that is not fully formed [74,75]. Bossu et al. [75] related that the levels of calcium and phosphorus were greater in two regions of the enamel of permanent teeth compared to that of deciduous teeth. 

Zamudio-Ortega et al. [76] reported similar outcomes in their study on deciduous teeth. Their analysis involved SEM, EDS, and XRD, which is comparable to the methods used in the present study, except for XRD. They reported mean atomic percentages and Ca/P molar ratios of the deciduous enamel surface using EDS analysis, which align with the findings presented in Table 4. However, the Ca/P ratios in the current study are smaller since the mean Ca/P ratios were compared between groups in versus the mentioned study. Although the same trace elements were examined, their reported values were higher than those observed in this study, as indicated in Table 5. The variation in Ca and P values could be attributed to factors such as the specific region where the teeth were collected, differences between individual teeth, variations in type, age, and ethnicity of the number of teeth analyzed, as well as differences in research methodologies [76].

Vitiello et al. [10] revealed in their EDS analysis the chemical changes associated with demineralization and remineralization; they noted a decrease in calcium and phosphate concentrations after demineralization, confirming the expected mineral loss. However, their study’s results showed significantly higher values compared to those reported by Wang et al. [77]. This discrepancy is attributed to their use of phosphoric acid alone for demineralization without employing pH cycling and the fact that they analyzed enamel, which has a higher mineral content than dentine, says Vitiello et al. [10]. Both these studies evaluated their research on permanent enamel and dentine, compared to this particular study that included only temporary enamel undergoing the laser treatment protocol listed in the materials and methods. The difference in the Ca/P ratio between permanent and primary enamel can be attributed to several factors. Primary enamel typically has a lower Ca/P ratio due to its less advanced mineralization and higher organic matrix content compared to permanent enamel. This means that primary enamel is less mineralized overall, resulting in a lower ratio of calcium to phosphorus.

Additionally, the physiological phases of primary enamel—formation, maturation, and exfoliation—affect its mineral content. Primary enamel never reaches the same level of mineralization as permanent enamel, and as it undergoes wear and demineralization before being shed, the Ca/P ratio can decrease further. Thus, the temporary reduction in these minerals does not diminish fluoride’s overall benefits, as it continues to enhance enamel strength and protection [78].

It can be observed from the surface morphology evaluation conducted after 980 nm diode laser treatment and after the pH cycle that no significant impacts were seen on the examined enamel region, and no fissures or fractures were triggered. We can conclude that diode lasers can offer a non-invasive, pain-free, and nearly safe treatment option based on the scanning electron microscopy (SEM) results that are consistent with those of Umana et al. [79] and Nandkumar et al. [80]. These studies demonstrated that 980 nm and 810 nm diode lasers at 0.8 W and 1 W, respectively, do not harm the enamel and dentin surface.

This study investigated the impact of three types of low-light laser radiation treatments, namely at wavelengths of 980 nm, 808 nm, and 450 nm, on dental hard tissue. This study’s findings revealed that all treatments had an influence on the surface morphological and chemical composition of the primary enamel hard tissue. The most significant enhancement in the chemical composition of enamel was achieved using laser diode irradiation of 808 nm by itself, where both calcium (Ca) and phosphorus (P) had an increase in the weight percentage, and when combining the laser and MI-varnish-fluoride together. For the 980 nm laser diode combined, the percentages of Ca and P also increased, resulting in an enhanced resistance to acid. Additionally, in the low diode laser 450 nm radiation, the 450 nm plus-MI varnish fluoride and the 808 nm plus the MI fluoride varnish treatments, even though the Ca percentage decreased, the P percentages increased significantly. The Ca/P ratio changes when enamel is treated with different irradiations due to the complex interplay of photothermal and photochemical effects, which vary with wavelength. The Ca/P ratio is a critical indicator of the enamel’s mineral quality. Changes in this ratio suggest alterations in the chemical composition of the enamel, possibly due to selective dissolution, precipitation, or transformation of calcium or phosphate phases [59,81]. The heating effects induced by 980 nm and 808 nm wavelengths may facilitate the recrystallization of hydroxyapatite, thereby increasing crystal order and density and potentially altering the surface composition and Ca/P ratio, while the higher energy photons at 450 nm could trigger photochemical reactions, including the generation of reactive oxygen species (ROS), leading to surface oxidation, demineralization, and subsequent variations in the Ca/P ratio [59,81,82]. There is a correlation between the amount of carbon (C) in teeth and several variables, including the hypoplastic condition of the enamel, food consumption, caries susceptibility, and enamel maturity [33]. Enamel becomes more resistant to demineralization as a result of this decrease in carbon concentration. Carbon reduction can substitute hydroxyl or phosphorus radicals in biological apatite crystals, resulting in a less soluble phase [83]. The findings of the current study relate to the previously mentioned research.

Due to their minimal side effects, diode light lasers prove to be a great asset in pediatric and preventive dentistry. Specialists should consider using low-light diode laser treatment to enhance the effectiveness of their dental treatment. The best ways to strengthen the structural resistance against solubility processes require more research to confirm the functions of laser settings.

It is necessary to conduct more research on this matter since the irradiation with low-laser diodes has the potential to inhibit demineralization and increase remineralization to a similar extent as fluoride therapy without causing any negative changes to the structure of the tooth enamel [72].

It can be concluded that laser radiation affects the chemical composition of enamel and that there are differences between the effects of the three different wavelengths on enamel surface morphology after the data were interpreted statistically. The null hypotheses formed at the beginning of the study were rejected, and the research hypotheses were accepted.

With respect to the study’s limitations, the following points should be noted: choosing primary teeth with the same chemical structure and the same exfoliation stage or, at best, a comparable concentration of chemical components is nearly impossible. Additionally, the surface morphology differed between samples. All these elements affected the study’s findings because of the enamel’s calcification, maturity, and density. Future studies might benefit from using a significantly greater number of samples to demonstrate the statistical significance of the variations in chemical composition and changes in enamel microhardness. Further in vitro and in vivo investigations using other methods of evaluation are required to assess the impact of 808 nm, 980 nm, and 450 nm laser diodes on pedodontics and preventive dentistry.

## 5. Conclusions

The study investigated the effects of 980 nm, 808 nm, and 450 nm low-light laser radiation on dental enamel, finding that these treatments, particularly when combined with MI-fluoride varnish, enhanced enamel smoothness and increased calcium (Ca) and phosphorus (P) levels, improving acid resistance. Due to their minimal side effects, diode lasers are valuable in pediatric and preventive dentistry, and specialists should consider incorporating them to enhance treatment outcomes. However, further research is needed to optimize laser settings for strengthening enamel’s structural resistance to solubility.

## Figures and Tables

**Table 1:** Brief summary of the eight groups and treatment applied to each group.

Group (G)	1	2	3	4	5	6	7	8
**FV-Varnish**	-	**X**	-	-	-	-	-	-
**L 980 nm**	-	-	**X**	-	-	**X**	-	-
**L 808 nm**	-	-	-	**X**	-	-	**X**	-
**L 450 nm**	-	-	-	-	**X**	-	-	**X**
**FV-After Laser**	-	-	-	-	-	**X**	**X**	**X**

**Table 2:** DIAGNOdent^®^ readings at initial scanning and after day 7 of remineralization among study groups.

Group	Pre-TX Mean	Day 7 Remin Mean	Mean Difference	*t*-Test *p*-Value
Control	2.83	4.75	1.92	<0.001 ***
Varnish	3.67	4.08	0.42	0.053
980 nm	3.33	2.83	−0.50	0.052
808 nm	3.33	2.83	−0.50	0.026 *
450 nm	3.00	2.50	−0.50	0.026 *
980 nm + Varnish	5.08	3.25	−1.83	<0.001 ***
808 nm + Varnish	3.92	3.00	−0.92	0.019 *
450 nm + Varnish	5.08	3.42	−1.67	<0.001 ***

The asterisks indicate the level of significance, with * for 0.01 < *p* < 0.05 and *** for *p* < 0.001.

**Table 3:** Tukey HSD results for mean values of EDX weight percentage values between groups.

Comparison	Mean Difference	Adjusted *p*-Value	Significance
450 nm vs. 450 nm + varnish	−1.1667	0.018	*
450 nm vs. control	2.4167	<0.001	**
450 nm + varnish vs. control	3.5833	<0.001	**
450 nm + varnish vs. varnish	2.0833	<0.001	**
808 nm vs. 808 nm + varnish	−0.4167	0.919	-
808 nm vs. control	2.4167	<0.001	**
808 nm + varnish vs. control	2.8333	<0.001	**
808 nm + varnish vs. varnish	1.3333	0.003	**
980 nm vs. 980 nm + varnish	−1.3333	0.003	**
980 nm vs. control	2.4167	<0.001	**
980 nm + varnish vs. control	3.75	<0.001	**
980 nm + varnish vs. varnish	2.25	<0.001	**
control vs. varnish	−1.5	<0.001	**

The asterisks indicate the level of significance, with * for 0.01 < *p* < 0.05 and ** for 0.001 < *p* < 0.01.

**Table 4:** Comparative weight percentage (Wt%) of mineral elements between study groups; TX—after treatment; PH—after pH cycle remineralization (both ANOVA *p* < 0.001).

Element	Control Group		Varnish Group		980 nm		808 nm		450 nm		980 nm + Varnish		808 nm + Varnish		450 nm + Varnish	
	Wt% TX	Wt% PH	Wt% TX	Wt% PH	Wt% TX	Wt% PH	Wt% TX	Wt% PH	Wt% TX	Wt% PH	Wt% TX	Wt% PH	Wt% TX	Wt% PH	Wt% TX	Wt% PH
	*Mean ± SD*															
**Ca**	**8.48** ± 1.16	**9.65** ± 1.32	**10.65** ± 1.76	**8.13** ± 1.35	**10.74** ± 1.58	**10.08** ± 1.49	**5.18** ± 0.74	**8.86** ± 1.26	**13.20** ± 1.91	**7.27** ± 1.05	**9.36** ± 1.68	**12.21** ± 2.19	**8.27** ± 1.42	**6.84** ± 1.18	**8.67** ± 1.52	**6.38** ± 1.12
**P**	**11.43** ± 1.76	**14.12** ± 2.17	**14.20** ± 2.65	**12.68** ± 2.37	**10.76** ± 1.79	**12.96** ± 2.15	**6.78** ± 1.09	**10.5** ± 1.69	**13.44** ± 2.19	**14.39** ± 2.35	**9.85** ± 1.99	**15.29** ± 3.08	**9.86** ± 1.91	**11.26** ± 2.18	**9.26** ± 1.83	**13.46** ± 2.66
**C**	**22.1** ± 2.93	**13.27** ± 1.76	**16.85** ± 2.71	**15.98** ± 2.57	**23.72** ± 3.4	**13.08** ± 1.87	**24.7** ± 3.42	**12.07** ± 1.67	**13.88** ± 1.95	**15.54** ± 2.19	**23.04** ± 4.00	**14.96** ± 2.6	**26.17** ± 4.37	**13.57** ± 2.27	**27.22** ± 4.63	**15.59** ± 2.65
**O**	**54.27** ± 6.15	**60.69** ± 6.88	**54.51** ± 7.49	**60.91** ± 8.37	**49.29** ± 6.03	**61.08** ± 7.48	**59.17** ± 7.00	**66.29** ± 7.84	**56.79** ± 6.83	**59.89** ± 7.20	**54.83** ± 8.14	**54.7** ± 8.12	**52.22** ± 7.46	**65.5** ± 9.36	**52.51** ± 7.64	**59.91** ± 8.71
**Na**	**1.65** ± 0.12	**1.46** ± 0.10	**1.97** ± 0.17	**1.14** ± 0.10	**0.83** ± 0.06	**1.52** ± 0.12	**1.26** ± 0.09	**1.27** ± 0.10	**1.29** ± 0.10	**1.47** ± 0.11	**1.29** ± 0.12	**1.15** ± 0.11	**1.49** ± 0.13	**1.46** ± 0.13	**1.00** ± 0.09	**2.01** ± 0.19
**Mg**	**0.35** ± 0.03	**0.42** ± 0.03	**0.62** ± 0.06	**0.32** ± 0.03	**0.16** ± 0.01	**0.29** ± 0.03	**0.32** ± 0.03	**0.41** ± 0.04	**0.49** ± 0.04	**0.56** ± 0.05	**0.16** ± 0.02	**0.29** ± 0.03	**0.34** ± 0.04	**0.39** ± 0.04	**0.30** ± 0.03	**0.87** ± 0.09
**Al**	**0.56** ± 0.05	**0.12** ± 0.01	**0.56** ± 0.06	**0.34** ± 0.04	**0.85** ± 0.08	**0.40** ± 0.04	**0.38** ± 0.04	**0.18** ± 0.02	**0.28** ± 0.03	**0.27** ± 0.03	**0.38** ± 0.05	**0.58** ± 0.07	**0.17** ± 0.02	**0.24** ± 0.03	**0.15** ± 0.02	**0.66** ± 0.08
**Si**	**0.79** ± 0.04	**0.09** ± 0.00	**0.25** ± 0.02	**0.13** ± 0.01	**2.76** ± 0.16	**0.14** ± 0.01	**0.63** ± 0.04	**0.17** ± 0.01	**0.07** ± 0.00	**0.23** ± 0.01	**0.15** ± 0.01	**0.21** ± 0.01	**0.25** ± 0.02	**0.29** ± 0.02	**0.20** ± 0.01	**0.46** ± 0.03
**Cl**	**0.38** ± 0.02	**0.16** ± 0.01	**0.39** ± 0.02	**0.37** ± 0.02	**0.28** ± 0.01	**0.45** ± 0.02	**0.18** ± 0.01	**0.26** ± 0.01	**0.29** ± 0.01	**0.38** ± 0.02	**0.38** ± 0.02	**0.61** ± 0.03	**0.39** ± 0.02	**0.45** ± 0.02	**0.35** ± 0.02	**0.65** ± 0.03

**Table 5:** Tukey HSD results for Ca/P ratios between groups.

Comparison	Mean Difference	Adjusted *p*-Value	Significance
***Ca/P Ratio TX***			
450 nm vs. 808 nm	−0.4295	0.0127	*
***Ca/P Ratio pH***			
450 nm vs. 808 nm	0.3333	0.0055	**
450 nm vs. 980 nm	0.2857	0.0265	*
450 nm vs. 980 nm + varnish	0.3160	0.0099	**
450 nm + varnish vs. 808 nm	0.3610	0.0021	**
450 nm + varnish vs. 980 nm	0.3134	0.0108	**
450 nm + varnish vs. 980 nm + varnish	0.3437	0.0038	**

This table summarizes just the significant findings (just TRUE results for null hypothesis rejection). The asterisks indicate the level of significance, with * for 0.01 < *p* < 0.05 and ** for 0.001 < *p* < 0.01.

## Data Availability

The original contributions presented in the study are included in the article, further inquiries can be directed to the corresponding author.
